# Association of sST2 with liver function in patients with acute heart failure related hospitalization

**DOI:** 10.3389/fcvm.2026.1781835

**Published:** 2026-09-03

**Authors:** Zhenyi Liu, Ying Wang, Yuanyuan Song, Xiuyong Yu, Hanxin Yao

**Affiliations:** 1 Department of Clinical Laboratory, The First Hospital of Jilin University, Changchun, China; 2 Department of Clinical Laboratory, BaiShan Central Hospital, Baishan, China

**Keywords:** acute heart failure, AST, DBIL, liver injury, NT-proBNP, sST2

## Abstract

**Objective:**

This study aimed to investigate the association between serum soluble ST2 (sST2) and liver injury biomarkers in patients with acute heart failure (AHF), and to evaluate the potential value of sST2 in identifying liver injury in AHF.

**Methods:**

A total of 134 patients with AHF (with or without liver injury) were prospectively enrolled, along with 187 age- and sex-matched healthy controls. Serum sST2 were measured within 24 h of hospital admission, along with comprehensive clinical and laboratory assessments. Univariate and multivariate linear regression analyses were performed to explore the relationships between sST2 and liver function parameters. ROC curve analysis was used to evaluate the discriminative ability of sST2 for liver injury.

**Results:**

Univariate analysis showed that sST2 had weak but statistically significant correlations with NT-proBNP (*r* = 0.20), TBIL (*r* = 0.26), and GGT (*r* = 0.19), moderately weak correlations with DBIL (*r* = 0.32) and ALP (*r* = 0.35), and strong correlations with AST (*r* = 0.71) and ALT (*r* = 0.55) (all *P* < 0.05). No significant association was observed between sST2 and cTnI (*r* = 0.07, *P* = 0.485). In multivariate regression, NT-proBNP (β = 0.001, *P* = 0.009), AST (β = 0.230, *P* < 0.001), and DBIL (β = 1.028, *P* = 0.003) were identified as independent predictors of sST2. Patients with liver injury had significantly higher sST2 levels (*P* = 0.002). ROC analysis demonstrated that sST2 predicted liver injury with an AUC of 0.691, which was superior to NT-proBNP (AUC = 0.545). The combined model of sST2, AST, and DBIL yielded an AUC of 0.905, with a sensitivity of 85.2% and a specificity of 85.0%.

**Conclusion:**

Serum sST2 is strongly associated with liver injury markers, particularly AST and DBIL, in patients with AHF. Elevated sST2 is associated with underlying hepatic dysfunction and may serve as a potential biomarker linking cardiac decompensation and liver impairment. Although sST2 alone has limited sensitivity for liver injury, a combined panel of sST2, AST, and DBIL provides excellent diagnostic performance. This combined approach may serve as a useful tool for risk stratification and early identification of cardiogenic liver injury.

## 1 Introduction

Heart failure (HF) is a complex and prevalent clinical syndrome characterized by impaired cardiac pumping function resulting from structural and/or functional abnormalities of the heart. It often represents a severe manifestation or the end stage of various cardiovascular diseases. According to the universal definition and classification of HF, HF is defined as “a clinical syndrome with symptoms and/or signs caused by a structural and/or functional cardiac abnormality and corroborated by elevated natriuretic peptide levels and/or objective evidence of pulmonary or systemic congestion” (1). Common symptoms include dyspnea, fatigue, and edema or congestion due to fluid retention, which represent the major pathophysiological manifestations that define HF in the context of cardiac dysfunction. Natriuretic peptides (NPs), such as brain natriuretic peptide (BNP) and N-terminal pro-brain natriuretic peptide (NT-proBNP), protect against pathological salt-water retention (edema/congestion) and are therefore strong mechanism-based biomarkers for HF. The prevalence of HF is estimated at 1%–2% among adults and exceeds 10% in individuals aged 70 years or older (2). In clinical practice, BNP and NT-proBNP are the most commonly used biomarkers for HF. They are considered gold standards for diagnosis, risk stratification, and guiding clinical management. However, NPs have well-recognized limitations, as their circulating concentrations can be affected by factors such as renal dysfunction, age, obesity, atrial fibrillation, and a range of cardiac and non-cardiac conditions unrelated to HF (3).

HF and liver disease frequently coexist. This is partly due to overlapping systemic risk factors, such as alcohol abuse, drug toxicity, chronic inflammation, autoimmunity, and infections. Complex bidirectional cardiohepatic interactions also play a role. HF can lead to hepatic congestion and hypoperfusion. This contributes to liver dysfunction, which in turn worsens prognosis and complicates HF management. Conversely, primary liver disease may independently induce cardiac dysfunction and even precipitate HF in patients without preexisting cardiovascular abnormalities (4, 5). The molecular mechanisms underlying hepatic injury under such conditions involve oxidative stress, mitochondrial dysfunction, and inflammatory cascades, as extensively reviewed in the context of hepatic ischemia reperfusion injury (6).

sST2 has gained attention as a biomarker for liver injury and inflammation. Elevated sST2 levels have been linked to poor prognosis in acute-on-chronic hepatitis B liver failure (7), advanced fibrosis in chronic hepatitis C (8), and increased disease severity in alcoholic liver disease (9). These findings highlight the potential of sST2 for noninvasive assessment of liver damage and systemic inflammation.

Soluble growth stimulation expressed gene 2 protein (sST2), a circulating isoform of the ST2 receptor, has emerged as one of the most promising biomarkers for risk stratification in HF (3). ST2 belongs to the interleukin-1 receptor superfamily and exerts its biological effects through binding with its specific ligand, interleukin-33 (IL-33). The interaction between IL-33 and membrane-bound ST2 exerts cardioprotective effects, while sST2 acts as a decoy receptor that inhibits this pathway, thereby reflecting myocardial stress, inflammation, and fibrosis (10). sST2 was first recognized as a prognostic biomarker in HF and was incorporated into the 2013 ACC/AHA guidelines for the assessment of cardiac function and prognosis. The updated 2022 AHA/ACC/HFSA Guideline for the Management of Heart Failure further recommends sST2 as part of the biomarker panel for risk stratification, providing additive prognostic value beyond natriuretic peptides such as BNP and NT-proBNP (11). Recent evidence has further extended the utility of sST2 to the emergency department setting, where it provides valuable prognostic information for patients with acute heart failure (AHF) and acute coronary syndrome, supporting risk stratification and therapeutic decision-making (12). Unlike natriuretic peptides, sST2 levels are less affected by age, renal function, body mass index, and atrial fibrillation, which enhances its clinical utility in complex patient populations. Moreover, growing evidence supports the diagnostic and/or prognostic utility of sST2 not only in HF, but also in a range of other conditions, including myocardial infarction, chronic kidney disease, sepsis, trauma, and other cardiovascular diseases (10, 13, 14). These characteristics position sST2 as a valuable tool in the multidimensional assessment of cardiovascular risk.

Despite its growing relevance in both cardiac and hepatic disorders, the association between sST2 and other clinical biomarkers—particularly in patients with HF—remains incompletely understood. Therefore, the present study aimed to evaluate the potential value of sST2 in identifying liver injury in this population by investigating the relationship between serum sST2 levels and liver function indicators, as well as key cardiac biomarkers including NT-proBNP and troponin I, to better elucidate the multifactorial pathophysiology underlying cardiohepatic interactions.

## 2 Material and methods

### 2.1 Study population

**Figure 1:**
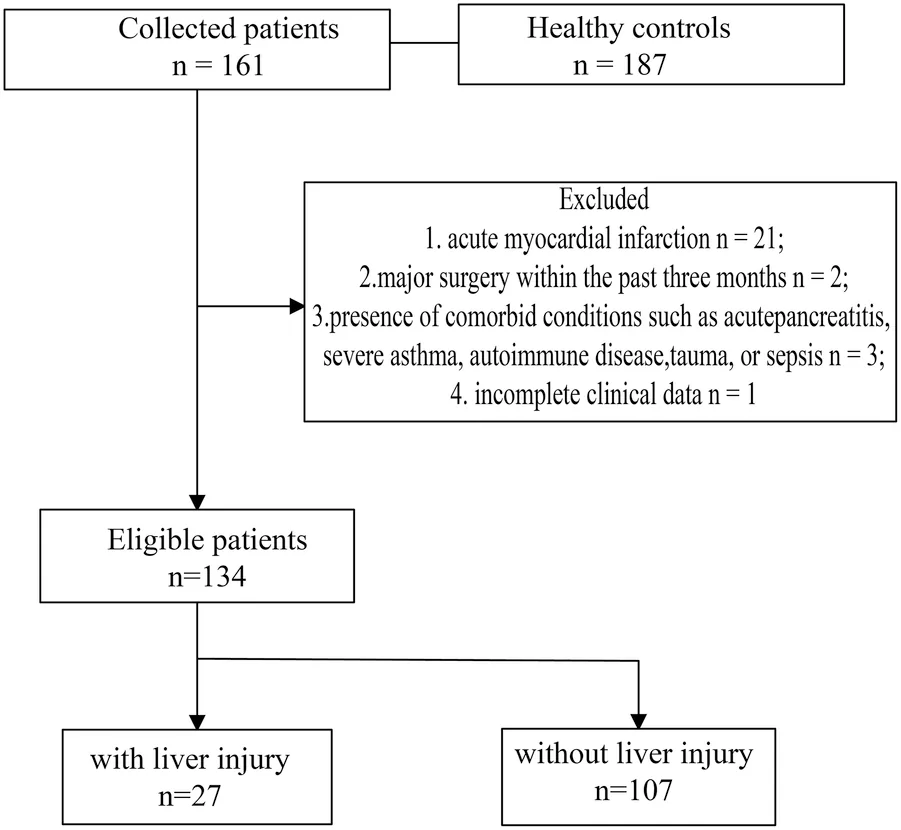
Flowchart of patient enrollment and exclusion. A total of 161 patients with suspected acute heart failure were screened, of whom 134 met the inclusion criteria and were enrolled. Additionally, 187 age- and sex-matched healthy controls were recruited concurrently. Enrolled patients were classified into those with (*n* = 27) and without (*n* = 107) liver injury based on predefined diagnostic criteria.

This study recruited 161 patients admitted to Jilin University First Hospital of Bethune from January 2023 to June 2024. The main reason for hospitalization in these patients was AHF or acute exacerbation of chronic HF. Patients were eligible for inclusion if they had a confirmed diagnosis of HF based on the Chinese Guidelines for the Diagnosis and Treatment of Heart Failure (2024 edition) (15), and were 18 years of age or older. Exclusion criteria included incomplete clinical data, a recent acute myocardial infarction, major surgery within the past three months. Comorbid conditions such as acute pancreatitis, severe asthma, autoimmune disease, trauma, or sepsis also led to exclusion. Finally, 134 patients were registered (Figure 1). In addition, a healthy control group comprising 187 age- and sex-matched volunteers was recruited concurrently, and baseline serum sST2 levels were measured using the same assay protocol.

Patients with liver injury were identified based on clinical and laboratory data, according to the diagnostic criteria for adult acute liver injury (ALI) outlined in the Expert Consensus on Diagnosis and Treatment of Acute Liver Injury in the Emergency Setting (16). ALI refers to a clinical condition characterized by an abrupt deterioration of liver function occurring within two weeks in individuals without pre-existing liver disease or with stable chronic liver disease. It results from direct or indirect exposure to hepatotoxic insults, including infections, trauma, drugs, toxins, or other physical and chemical factors. Patients commonly present with nonspecific symptoms such as fatigue, loss of appetite, nausea, vomiting, upper abdominal discomfort, and jaundice, which may be accompanied by physical findings such as icteric sclera or skin, hepatomegaly, or ascites. The diagnosis of ALI was based on both clinical manifestations and laboratory abnormalities. Laboratory tests needed to meet one or more of the following criteria: (1) alanine aminotransferase (ALT) ≥ 2 × upper limit of normal (ULN), i.e., ≥ 80 U/L (normal reference range 0–40 U/L); (2) alkaline phosphatase (ALP) > 150 U/L (normal reference range 40–150 U/L for adults), total bilirubin (TBIL) > 17.1 μmol/L (normal reference range 3.4–17.1 μmol/L), or international normalized ratio (INR) > 1.2 (normal reference range 0.8–1.2). All patients with suspected liver injury underwent systematic evaluation to exclude non-cardiac etiologies. Specifically, we reviewed each patient's medication history (excluding drug-induced liver injury), alcohol use history (excluding alcoholic hepatitis), viral hepatitis serological markers (excluding hepatitis B and C), and autoimmune liver disease work-up when clinically indicated. Patients with evidence of acute viral hepatitis, drug-induced liver injury, alcoholic hepatitis, autoimmune hepatitis, or other primary liver diseases were excluded from the analysis.

The study was approved by the Ethics Committee of the First Hospital of Jilin University (N0. 2024-643). All participants provided general informed consent authorizing the use of their clinical data and biological samples for relevant research. All procedures involving human participants were conducted in accordance with the ethical standards of the institutional and/or national research committee, and with the Declaration of Helsinki and its later amendments or comparable ethical standards. We confirm that all methods were performed in accordance with the relevant guidelines and regulations.

### 2.2 Blood samples and biomarker analysis

Blood samples were collected within 24 h of patient admission. Blood samples from healthy controls were collected simultaneously. Fresh serum samples were used for sST2 measurement. The samples were centrifuged at 2,000× *g* for 10 min at room temperature to separate serum. Serum sST2 levels were measured using the Pylon ST2 assay on the Pylon IRIS immune analyzer (ET healthcare Inc., Suzhou, China). The assay's linear range is 1–1,000 ng/mL, and the intra- and inter-assay coefficients of variation are ≤ 10%. Daily quality control materials were used to monitor the performance of the ST2 assay throughout the testing process. Calibration and quality control were performed using the kit-matched calibrators and control materials according to the manufacturer's specifications. Additional clinical and laboratory data were obtained from patient records and the hospital information system. These included: N-terminal pro-brain natriuretic peptide (NT-proBNP), cardiac troponin I (cTnI), estimated glomerular filtration rate (eGFR), low-density lipoprotein cholesterol (LDL-C), high-density lipoprotein cholesterol (HDL-C), white blood cell count (WBC), neutrophil count (NEU), procalcitonin (PCT), glycated hemoglobin (HbA1c), glucose (GLU), gamma-glutamyl transferase (GGT), aspartate aminotransferase (AST), alanine aminotransferase (ALT), alkaline phosphatase (ALP), total bilirubin (TBIL), direct bilirubin (DBIL), body mass index (BMI), and left ventricular ejection fraction (LVEF).

### 2.3 Statistical analysis

All statistical analyses were performed using MedCalc (version 20.0.10) and GraphPad Prism (version 10.1.2). Continuous variables were first tested for normality. Data following a normal distribution were expressed as mean ± standard deviation (SD) and compared among groups using one-way analysis of variance (ANOVA). Data that were not normally distributed were presented as median with interquartile range (IQR) and compared using the Kruskal–Wallis test. For comparisons between two groups, the independent samples t-test was applied for normally distributed data, and the Mann–Whitney U test for non-normally distributed data. Categorical variables were expressed as frequencies (percentages) and compared using the chi-square (*χ*^2^) test. The Mann–Whitney U test was used for comparisons between two groups, while the Kruskal–Wallis test was applied for comparisons among more than two groups. The influencing factors of sST2 were evaluated using univariate and multivariate linear regression analyses. Multivariate linear regression analysis was conducted using the enter method, whereby all variables with *P* < 0.10 in univariate analysis were entered simultaneously into the model. Multicollinearity was assessed by calculating variance inflation factors (VIFs), with a VIF > 5 indicating potential multicollinearity. Receiver operating characteristic (ROC) curve analysis was performed to assess the predictive value of sST2 and NT-proBNP for liver injury in patients with AHF.

## 3 Results

### 3.1 The characteristics of the study population

The study included 134 patients with AHF, with a median age of 64.5 years (IQR: 51.0–74.0), of whom 76 (56.7%) were male. According to the the New York Heart Association (NYHA) classification, the majority were in advanced stages: 23.9% in class III and 68.7% in class IV. Common comorbidities were arrhythmia (58.2%), diabetes mellitus (41.3%), acute coronary syndrome (45.5%), renal dysfunction (35.1%), and hypertension (27.6%), while 12.7% had venous or intracardiac thrombosis. Overall, 20.1% (27/134) of patients had liver injury. Compared with those without liver injury, affected patients were younger (55.0 vs. 66.0 years old, *P* = 0.038), had lower prevalence of diabetes mellitus (22.2% vs. 44.9%, *P* = 0.033), but higher prevalence of arrhythmia (77.8% vs. 53.3%, *P* = 0.022) and venous/intracardiac thrombosis (25.9% vs. 9.3%, *P* = 0.021). Regarding baseline biomarkers, patients with liver injury had higher levels of uric acid (577.0 vs. 433.0 μmol/L, *P* = 0.003), WBC (8.79 vs. 7.08 10⁹/L, *P* = 0.049), NEU (6.16 vs. 4.73 10⁹/L, *P* = 0.014), and PCT (0.23 vs. 0.10 ng/mL, *P* = 0.034). Moreover, liver function markers were markedly elevated in the liver injury group, including GGT, AST, ALT, ALP, TBIL, and DBIL (all *P* < 0.001). No significant differences were observed in creatinine, eGFR, lipid profile, HbA1c, glucose, troponin I, or LVEF between the two groups. Baseline characteristics are summarized in Table 1.

**Table 1:** Patient characteristics and laboratory test results.

Characteristics	Overall (*n* = 134)	Without liver injury (*n* = 107)	Liver injury (*n* = 27)	*P* value
Age (years), median (IQR)	64.50 (51.00, 74.00)	66.00 (54.0, 74.0)	55.00 (43.75, 66.00)	0.038
Men, *n* (%)	76 (56.7)	58 (54.2)	18 (66.7)	0.245
BMI (kg/m^2^), median (IQR)	24.8 (23.20, 27.50)	24.5 (22.90, 27.23)	26.8 (23.40, 29.50)	0.227
NYHA class, *n* (%)				0.084
I	2 (1.5)	2 (1.9)	0 (0.0)	
II	8 (6.0)	5 (4.7)	3 (11.1)	
III	32 (23.9)	30 (28.0)	2 (7.4)	
IV	92 (68.7)	70 (65.4)	22 (81.5)	
Type of HF, *n* (%)				0.143
HFrEF	78 (58.2)	63 (58.9)	15 (55.6)	
HFmrEF	17 (12.7)	11 (10.3)	6 (22.2)	
HFpEF	27 (20.1)	24 (22.4)	3 (11.1)	
Unknown	12 (9.0)	9 (8.4)	3 (11.1)	
Comorbidities, *n* (%)				
Hypertension	37 (27.6)	31 (29.0)	6 (22.2)	0.485
Diabetes mellitus	54 (41.3)	48 (44.9)	6 (22.2)	0.033
Valvular disease	27 (20.1)	23 (21.5)	4 (14.8)	0.441
Renal dysfunction	47 (35.1)	37 (34.6)	10 (37.0)	0.812
Arrhythmia	78 (58.2)	57 (53.3)	21 (77.8)	0.022
Venous thrombosis/intracardiac thrombosis	17 (12.7)	10 (9.3)	7 (25.9)	0.021
Acute coronary syndrome	61 (45.5)	52 (48.6)	9 (33.3)	0.156
Stroke	7 (5.2)	7 (6.5)	0 (0.0)	0.174
Biomarkers (baseline), median (IQR)				
eGFR, mL/min/1.73 m^2^	70.75 (42.30, 89.40)	71.10 (45.05, 89.95)	68.70 (36.53, 85.80)	0.616
Creatinine, μmol/L	89.05 (69.80, 129.55)	83.60 (68.55, 124.18)	100.00 (73.35, 160.80)	0.243
Uric acid, μmol/L	462 (354.00, 600.50)	433.00 (342.00, 549.00)	577.00 (428.25, 737.00)	0.003
Cholesterol, mmol/L	4.07 (3.42, 4.84)	4.11 (3.43, 4.92)	4.05 (3.30, 4.47)	0.656
LDL-C,mmol/L	2.49 (2.09, 3.05)	2.49 (2.03, 3.07)	2.54 (2.18, 2.95)	0.75
HDL-C, mmol/L	0.94 (0.79, 1.17)	0.95 (0.80 1.18)	0.94 (0.76, 1.06)	0.519
WBC, 109/L	7.17 (5.80, 9.56)	7.08 (5.60, 9.03)	8.79 (6.33, 10.84)	0.049
NEU, 109/L	4.84 (3.93, 6.90)	4.73 (3.78, 6.40)	6.16 (4.50, 7.47)	0.014
PCT, ng/mL	0.11 (0.05, 0.20)	0.10 (0.05, 0.15)	0.23 (0.11, 0.46)	0.034
HBA1C, %	6.35 (6.10, 7.40)	6.30 (6.05, 7.55)	6.50 (6.10, 6.80)	0.828
GLU, mmol/L	6.39 (5.40, 7.96)	6.50 (5.40, 7.95)	6.36 (5.40, 8.21)	0.784
GGT, U/L	41.50 (28.30, 70.70)	39.80 (23.68, 59.38)	83.50 (42.70, 129.55)	<0.001
AST, U/L	26.05 (19.30, 48.30)	22.30 (17.50, 35.60)	66.50 (40.50, 359.65)	<0.001
ALT, U/L	22.70 (13.70, 49.90)	19.90 (12.45, 31.00)	70.60 (32.95, 246.18)	<0.001
ALP, U/L	79.80 (66.90, 98.40)	76.60 (64.08, 96.20)	92.20 (78.53,127.55)	<0.001
TBIL, μmol/L	15.85 (11.80, 29.00)	14.80 (11.30, 21.83)	37.60 (23.63, 53.75)	<0.001
DBIL, μmol/L	3.70 (2.20, 8.50)	3.20 (2.10, 4.88)	12.00 (7.20, 18.43)	<0.001
Troponin I, ng/mL	0.02 (0.01, 0.11)	0.02 (0.01, 0.11)	0.02 (0.01 0.10)	0.922
LVEF (%)	37.00 (29.0, 47.0)	37.00 (29.00, 48.00)	36.5 (29.00, 43.50)	0.682
NT-proBNP, pg/mL	4699.50 (1948.00, 11696.00)	4779.00 (1782.00, 11516.75)	4212.00 (3375.75, 11069.75)	0.469
sST2, ng/mL	26.50 (16.80, 50.20)	24.70 (15.28, 41.35)	47.90 (22.78, 184.00)	0.002

### 3.2 Univariate linear regression analysis of sST2 and other biomarkers

**Figure 2:**
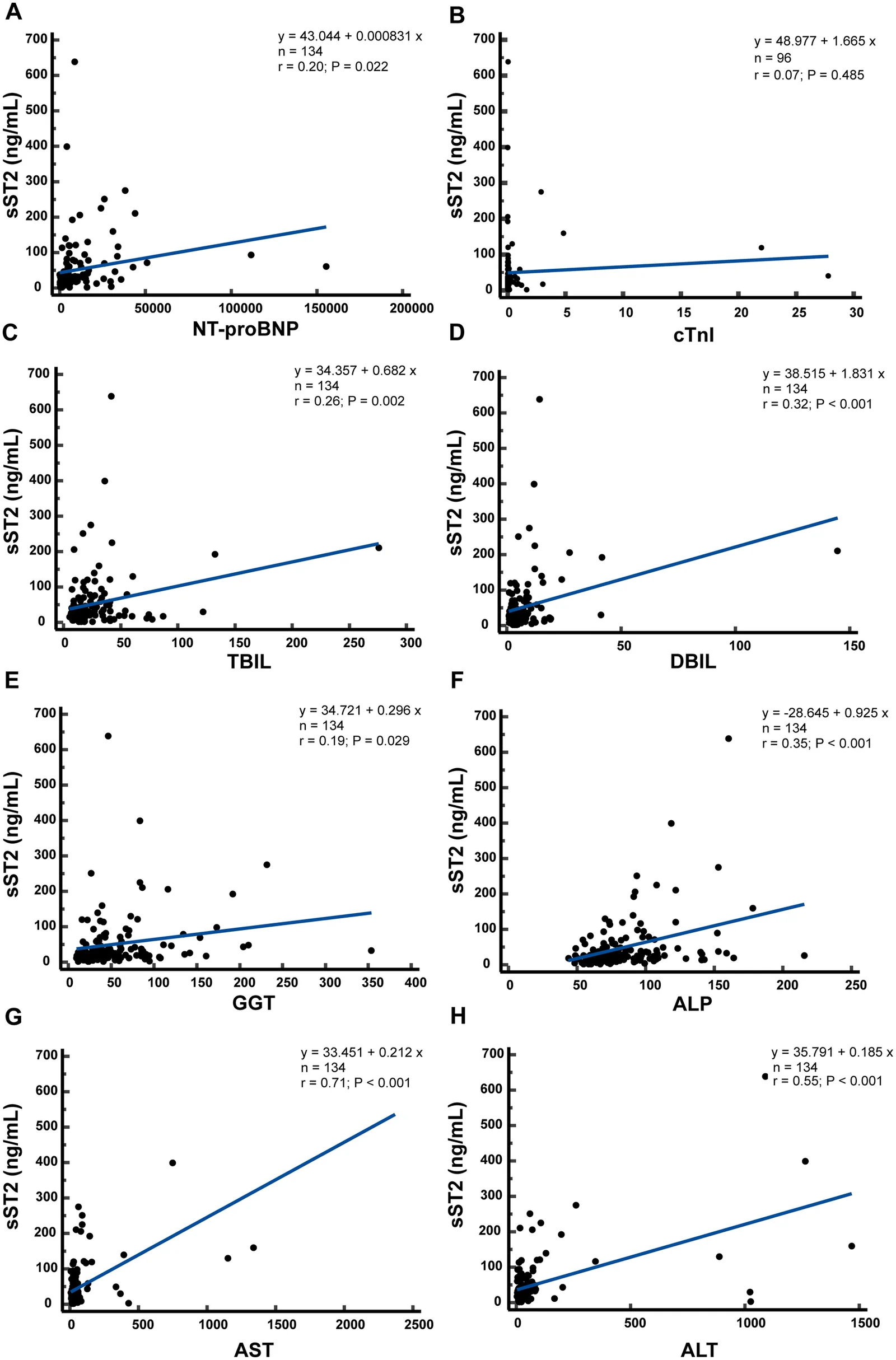
Correlation of serum sST2 with cardiac and liver function markers in HF patients. **(A,B)** Associations between sST2 and cardiac biomarkers NT-proBNP and cTnI. **(C–H)** Associations between sST2 and liver function parameters, including TBIL, DBIL, GGT, ALP, AST, and ALT. The solid lines represent linear regression fits.

We evaluated the influencing factors of serum sST2 levels using univariate linear regression analysis (Figure 2). The analysis demonstrated a weak but significant positive association between sST2 levels and the HF biomarker NT-proBNP (β = 0.001, *r* = 0.20, *P* = 0.022; Figure 2A), whereas no significant correlation was observed with the myocardial injury marker cTnI (β = 1.665, *r* = 0.07, *P* = 0.485; Figure 2B). Regarding liver function markers, sST2 levels showed a weak but significant positively associated with TBIL (β = 0.682, *r* = 0.26, *P* = 0.002; Figure 2C), a moderately weak positive correlation with DBIL (β = 1.831, *r* = 0.32, *P* < 0.001; Figure 1D), a weak but significant positive correlation with GGT (β = 0.296, *r* = 0.19, *P* = 0.029; Figure 2E), and a moderately weak positive correlation with ALP (β = 0.925, *r* = 0.35, *P* < 0.001; Figure 2F). Furthermore, sST2 levels were strongly positively associated with hepatocellular injury markers, including AST (β = 0.212, *r* = 0.71, *P* < 0.001; Figure 2G) and ALT (β = 0.185, *r* = 0.55, *P* < 0.001; Figure 2H). In addition, correlation analysis revealed that sST2 levels were significantly associated with the inflammatory markers neutrophil count (NEU: *r* = 0.225, *P* = 0.010) and procalcitonin (PCT: *r* = 0.268, *P* = 0.029), whereas no significant correlation was observed with white blood cell count (WBC: *r* = 0.080, *P* = 0.365) (Supplementary Table S1). These findings suggest that serum sST2 levels are associated with cardiac stress and hepatocellular injury in patients with AHF. Associations with cholestatic injury and systemic inflammatory responses were also observed. In contrast, further analyses revealed no significant linear associations between NT-proBNP and liver function markers, including TBIL, DBIL, GGT, ALP, AST, and ALT (Supplementary Figures S1A–F).

### 3.3 Multivariate linear regression analysis of factors influencing serum sST2 levels

Multiple linear regression analysis was performed to evaluate the influence of NT-proBNP, DBIL, GGT, AST, ALT, and ALP on serum sST2 levels. Spearman's rank correlation coefficient between TBIL and DBIL was 0.902, indicating a very strong positive correlation. When both TBIL and DBIL were entered simultaneously into the multivariate linear regression model, the variance inflation factor (VIF) for each exceeded 5 (TBIL VIF = 7.72, DBIL VIF = 7.645), confirming the presence of significant multicollinearity. To avoid this issue, we retained DBIL in the final model because it showed a stronger univariate correlation with sST2 (Figures 2C–E). The model yielded an R^2^ value of 0.584, indicating that approximately 58.4% of the variability in sST2 levels could be explained by these independent variables. Among them, NT-proBNP (β = 0.001), AST (β = 0.230), and DBIL (β = 1.028) were found to be statistically significant predictors of sST2 levels (*P* < 0.05 for all; Table 2). In contrast, GGT, ALT, and ALP did not significantly affect sST2.

**Table 2:** Multiple linear regression of sST2 with clinical and laboratory parameters.

Variables	Coefficient β	*P* value	VIF
ALP	0.113	0.524	1.467
ALT	−0.007	0.844	3.35
AST	0.226	<0.001	3.603
DBIL	1.366	<0.001	1.153
GGT	0.169	0.112	1.47
NT-proBNP	0.001	0.015	1.169
Age	0.186	0.58	1.433
NYHA class	−1.593	0.816	1.119
LVEF	−8.758	0.834	1.531
Hypertension	2.828	0.806	1.329
Diabetes mellitus	7.039	0.505	1.392
Valvular disease	−14.671	0.216	1.187
Renal dysfunction	−8.547	0.423	1.284
Arrhythmia	−8.435	0.443	1.481
Venous thrombosis/intracardiac thrombosis	12.968	0.385	1.286
Acute coronary syndrome	5.113	0.604	1.227
Stroke	−0.086	0.997	1.086

VIF, Report Yariance inflation factor.

### 3.4 Predictive effect of sST2 on liver injury

**Figure 3:**
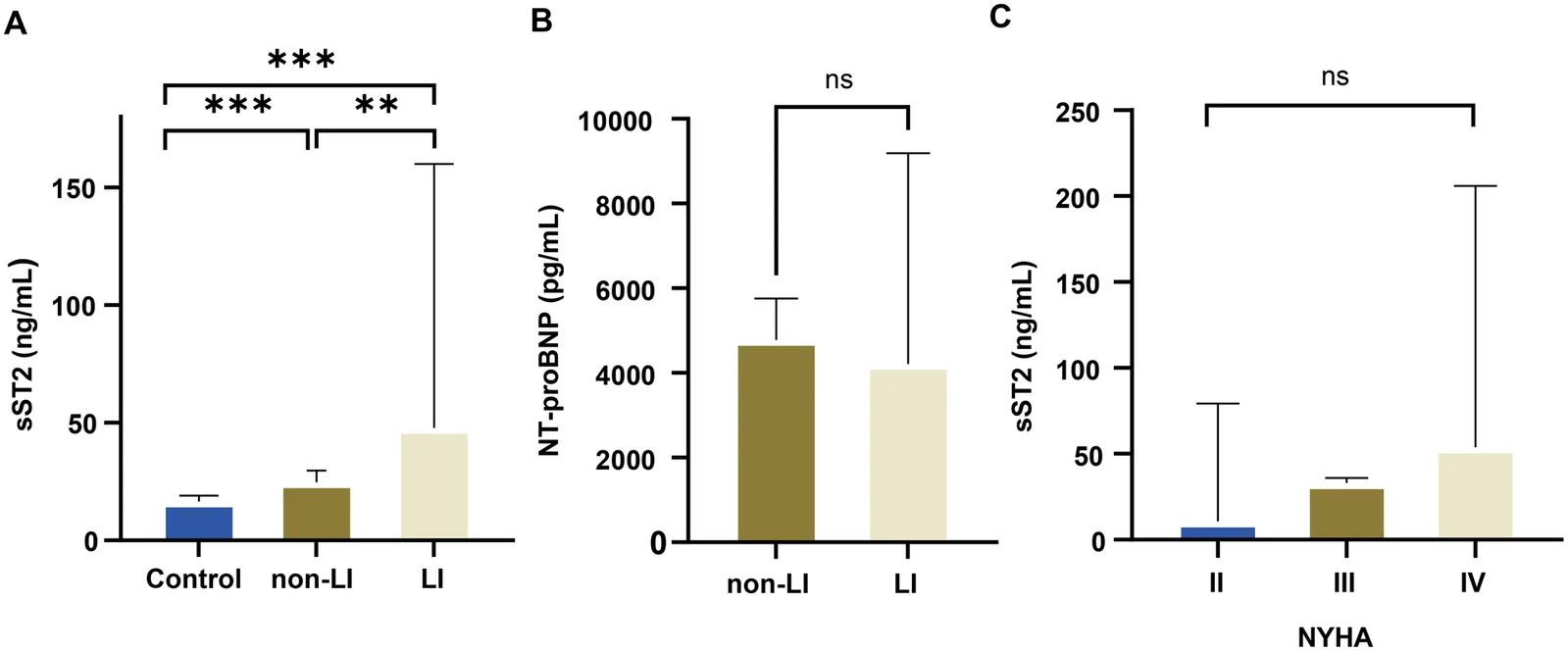
Comparison of serum sST2 and NT-proBNP levels in healthy controls and AHF patients with or without liver injury. **(A)** Serum sST2 levels in healthy controls (*n* = 187), AHF patients without liver injury (non-LI, *n* = 107), and AHF patients with liver injury (LI, *n* = 27). sST2 levels were significantly higher in AHF patients compared with healthy controls, and further elevated in those with liver injury (*P* < 0.001). **(B)** Serum NT-proBNP levels in AHF patients with and without liver injury. No significant difference was observed between the two groups (*P* = 0.473). **(C)** Serum sST2 levels across NYHA functional classes within the liver injury subgroup (*n* = 27). Although sST2 tended to increase with higher NYHA class, the difference did not reach statistical significance (*P* = 0.236). LI, liver injury; ns, not significant; **P < 0.01; ***P < 0.001.

Compared with healthy controls, serum sST2 levels were significantly increased in AHF patients, with the highest levels observed in those with liver injury (median [IQR]: healthy controls, 16.6 [11.4 −22.9] ng/mL; AHF without liver injury, 24.7 [15.28–41.35] ng/mL; AHF with liver injury, 47.9 [22.78–184.0] ng/mL; *P* < 0.001) (Figure 3A). In contrast, NT-proBNP levels did not differ significantly between the two groups (4,212.0 [3,375.8–11,069.8] vs. 4,779.0 [1,782.0–11,516.8] pg/mL; *P* = 0.473) (Figure 3B). Both groups showed markedly elevated NT-proBNP levels above the normal upper limit (125 pg/mL).

**Figure 4:**
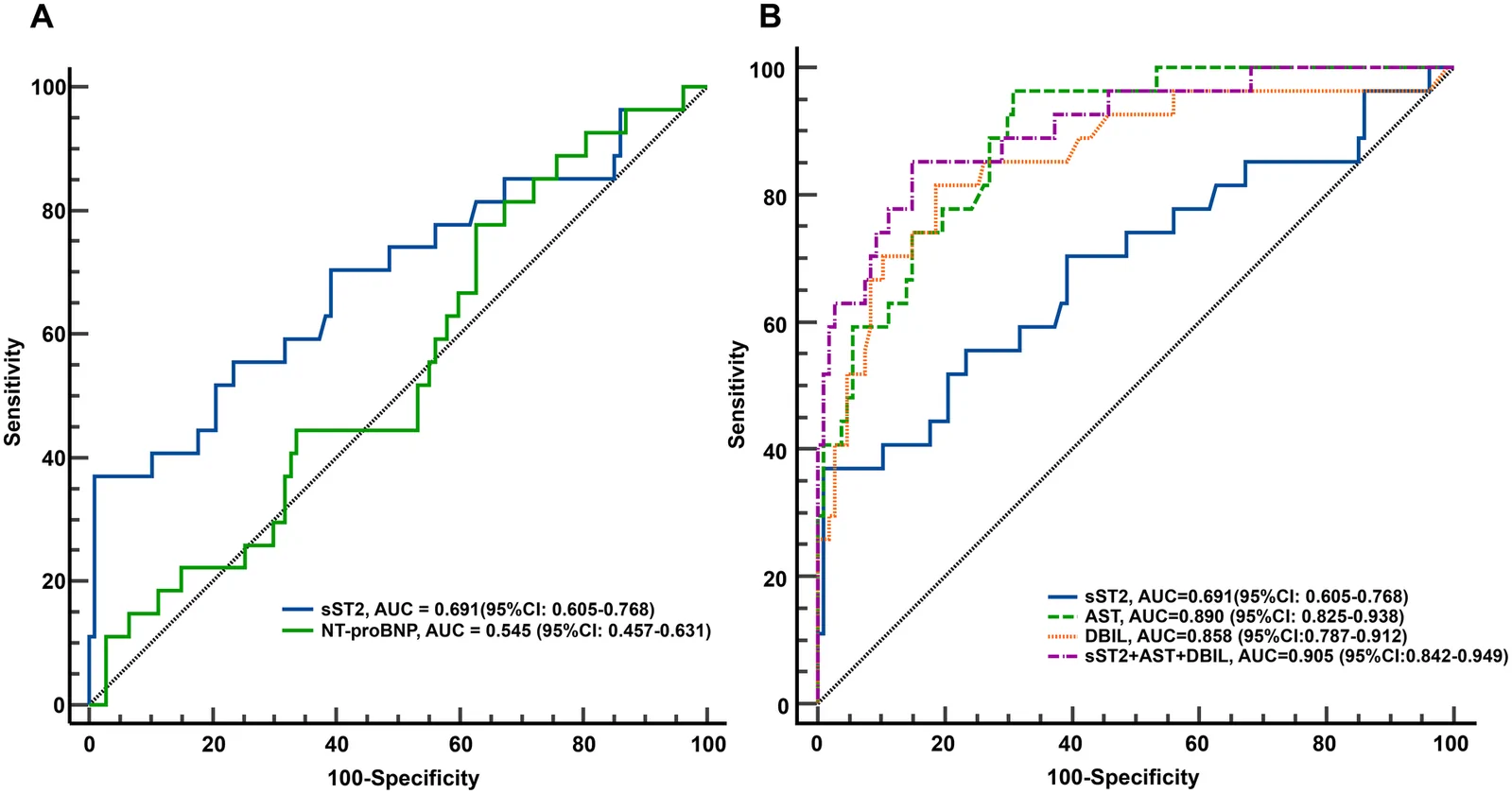
ROC curve analysis of biomarkers for identifying liver injury in AHF patients. **(A)** Comparison of sST2 and NT-proBNP for predicting liver injury. **(B)** Comparison of sST2, AST, DBIL, and their combined model (sST2 + AST + DBIL) for predicting liver injury.

ROC curve analysis was performed to evaluate the discriminative ability of sST2. For distinguishing AHF patients from healthy controls, sST2 yielded an AUC of 0.705 (95% CI: 0.651 −0.754; *P* < 0.001), with an optimal cutoff value of 28.7 ng/mL, providing 47.8% sensitivity and 90.4% specificity (Supplementary Figure S2). To assess the predictive value of sST2 and NT-proBNP for liver injury in AHF patients, ROC analysis was also performed. sST2 demonstrated an AUC of 0.691 (95% CI: 0.605–0.768; *P* = 0.003), with an optimal cutoff value of 121.0 ng/mL, providing 37.0% sensitivity and 99.1% specificity. In contrast, NT-proBNP showed significantly lower discriminatory ability [AUC = 0.545 (0.457–0.631); *P* = 0.006] (Figure 4A). To improve diagnostic efficacy, we constructed a combined logistic regression model incorporating sST2, AST, and DBIL. The combined model yielded an AUC of 0.905 (95% CI: 0.842–0.949), with a sensitivity of 85.2% and a specificity of 85.0%, which was superior to any single biomarker alone (sST2 alone: AUC 0.691; AST alone: AUC 0.890; DBIL alone: AUC 0.858) (Figure 4B; Supplementary Table S2).

## 4 Discussion

In this study, elevated AST, DBIL, and NT-proBNP levels were independently associated with increased serum sST2 in hospitalized patients with HF. Consistent with its established role as a cardiac stress marker, sST2 levels were positively correlated with NT-proBNP, confirming its reflection of myocardial stress and HF severity. In addition to its association with cardiac biomarkers, sST2 was significantly related to markers of hepatic dysfunction, including AST and DBIL, suggesting that sST2 elevation in AHF may also reflect systemic organ involvement. Notably, no significant correlations were observed between NT-proBNP and liver function biomarkers, whereas sST2 demonstrated superior discriminatory ability in identifying liver injury among AHF patients. Together, these findings support the dual role of sST2 as a biomarker of both cardiac stress and hepatic injury, underscoring the interconnected pathophysiology of HF and liver dysfunction.

The coexistence of HF and liver dysfunction is well established and is commonly referred to as the cardiohepatic syndrome. This bidirectional interaction between the heart and liver arises through several overlapping mechanisms. In the setting of HF, particularly right-sided or biventricular failure, passive hepatic congestion due to elevated central venous pressure is a major contributor to liver injury. Additionally, low cardiac output in advanced HF leads to hepatic hypoperfusion, resulting in ischemic hepatitis or “shock liver.” These hemodynamic disturbances provoke hepatocellular damage, bile stasis, and ultimately fibrosis if sustained over time (17, 18). Elevated liver enzymes, particularly AST and DBIL as observed in our study, are commonly interpreted as biochemical markers of hepatic congestion and cholestasis in this context.

Liver dysfunction in HF is not only a marker of advanced disease but also an independent predictor of poor outcomes. Large cohort studies have shown that abnormal liver biochemistry is associated with increased mortality and higher rates of hospital readmission in patients with HF (19, 20). This underscores the clinical relevance of incorporating liver function assessment into HF risk stratification. The present study adds to this body of evidence by demonstrating that sST2—a well-established biomarker of cardiac stress and remodeling—is closely related to indicators of hepatic dysfunction, thereby reinforcing the notion of multiorgan involvement in the progression of HF.

In the cardiovascular system, sST2 has emerged as a strong prognostic marker in both acute and chronic HF. It reflects myocardial fibrosis, cardiomyocyte stretch, and systemic inflammation, and is not significantly influenced by age, renal function, or body mass index—making it an attractive tool for risk stratification (21). Beyond its well-established role in cardiovascular risk stratification, sST2 has also been implicated in liver fibrosis and inflammation across various chronic liver diseases, including hepatitis B, hepatitis C, and alcoholic liver disease (7–9). Mechanistically, activation of hepatic stellate cells leads to IL-33 release, which engages the ST2 signaling pathway to promote fibrotic progression (22). However, these studies were conducted in patients with primary liver disease, not in the context of AHF. Using an experimental model of ischemic HF, Pascual-Figal and colleagues (23) found that sST2 increased in both lungs and myocardium, but no changes were observed in the liver or kidneys. ST2 immunostaining was intense in alveolar epithelium, and human type II pneumocytes secreted sST2 in response to mechanical strain. These findings suggest that the lungs are a relevant source of sST2 in HF, raising the possibility that elevated sST2 in AHF may primarily reflect pulmonary response to hemodynamic stress rather than hepatic injury *per se*. The discrepancy between their observations and our positive correlation of sST2 with AST and DBIL may be explained by differences in study design (animal model vs. human cohort), the severity of hepatic involvement, and the fact that their analysis focused on tissue expression rather than circulating sST2 correlations with liver injury markers. In our human AHF cohort, coexisting hepatic congestion and hypoperfusion likely contribute to sST2 elevation through systemic inflammation and end-organ damage, which may not be fully recapitulated in the experimental model. Several studies have demonstrated that elevated liver enzymes, including AST, ALT, and DBIL, are independent predictors of mortality and hospitalization risk in patients with HF, even in the absence of chronic liver disease (24). An increased AST/ALT ratio has also been associated with reduced left ventricular ejection fraction and elevated NT-proBNP levels, indicating a potential link between hepatic dysfunction and impaired cardiac performance (25). In the present study, we found that serum sST2 levels were independently associated with liver injury markers, specifically AST and DBIL, in multiple regression analyses. These findings suggest a potential interplay between sST2 and hepatic stress or inflammatory responses in the setting of cardiac dysfunction.

In AHF, reduced cardiac output leads to hepatic hypoperfusion, while elevated central venous pressure causes passive hepatic congestion (26). These two mechanisms predominantly affect the perivenular (zone 3) hepatocytes, which are rich in AST but relatively poor in ALT (27). Consequently, AST is more sensitive than ALT to hypoxic and congestive injury in this setting. In parallel, hepatic congestion impairs bile flow at the canalicular level, leading to an increase in conjugated DBIL without a proportional rise in total ALP because cholestasis is usually mild and not the dominant feature (28). By contrast, ALT and ALP are more reflective of hepatocellular necrosis (zone 1–2) or overt cholestasis, which are less characteristic of acute cardiogenic liver injury. Therefore, the independent association of sST2 with AST and DBIL—but not with ALT or ALP—is consistent with the pathophysiological pattern of hypoxic hepatitis and congestive hepatopathy in AHF. Oxidative stress and inflammatory cytokines mediate the activation of Kupffer cells and hepatic stellate cells (HSCs) induced by systemic inflammation and organ congestion in AHF (22). Activated HSCs release IL-33, which normally binds to membrane-bound ST2 (ST2L) to exert anti-fibrotic and tissue-protective effects. Indeed, experimental studies have demonstrated that activation of the IL-33/ST2 pathway promotes liver fibrosis by driving HSC activation, and that IL-33/ST2 signaling is required for the development of hepatic fibrosis (29). However, elevated levels of sST2 act as a decoy receptor, sequestering IL-33 and preventing its protective signaling by inhibiting IL-33/ST2L signaling and its beneficial effects (10). This imbalance may promote hepatic stellate cell activation, extracellular matrix deposition, and low-grade hepatic fibrosis even after the resolution of acute congestion. In line with this, serum sST2 levels have been shown to correlate with liver fibrosis stages in chronic liver disease and can identify patients with advanced fibrosis as a single serum marker (8). Moreover, sST2 elevation correlates with systemic inflammatory markers (NEU and PCT, Supplementary Table S1), suggesting that sST2 may serve as a bridge between systemic inflammation and liver injury in AHF. This role of sST2 in systemic inflammatory and fibrotic processes has been recognized across multiple disease contexts (30). These findings suggest that sST2 serves as a convergent biomarker of tissue injury and inflammation across organ systems. Its elevation in HF patients with liver injury may reflect not only cardiac strain, but also hepatic inflammation and fibrotic remodeling, potentially driven by IL-33/ST2 signaling. The decoy nature of sST2 blunts IL-33-mediated protective responses, such as tissue repair and anti-fibrotic effects (31). This phenomenon may be associated with the worsening of both cardiac and hepatic fibrosis in the setting of chronic systemic disease. Thus, the observed elevation of sST2 in HF patients with liver injury may reflect a complex interplay of hemodynamic stress, systemic inflammation, and impaired tissue repair.

Importantly, our finding that sST2 correlates with both cardiac (NT-proBNP) and hepatic (AST, DBIL) markers supports the concept of a synergistic cardiohepatic axis. Rather than sST2 acting independently on the heart and liver, we hypothesize that in AHF, elevated sST2 reflects a systemic response to combined hemodynamic and inflammatory insults, where the liver is a sensitive end-organ. In contrast to sST2, NT-proBNP levels did not show a significant correlation with liver injury markers in our cohort. Previous research has reported that NT-proBNP concentrations are markedly reduced in patients with HF complicated by liver disease, such as in non-alcoholic fatty liver disease and acute decompensated HF (32). This phenomenon may be related to decreased levels of angiotensin II in such patients, which could suppress NT-proBNP secretion. Similarly, in our study, NT-proBNP levels tended to be lower in AHF patients with liver injury, although the difference did not reach statistical significance. In contrast, sST2 levels were significantly elevated in AHF patients with liver injury and significantly related to markers of hepatic dysfunction. sST2 is a member of the interleukin-1 receptor family. It functions as a soluble decoy receptor for IL-33 and regulates inflammatory and fibrotic signaling in both cardiac and extracardiac tissues (30). Elevated sST2 reflects systemic inflammation, oxidative stress, and tissue remodeling—all of which are central to the pathophysiology of cardiohepatic syndrome. Therefore, sST2 may better capture the inflammatory and fibrotic components of liver injury associated with HF, explaining its stronger correlation with hepatic biomarkers compared with the hemodynamic stress marker NT-proBNP. Moreover, sST2 demonstrated a moderate discriminative ability for identifying liver injury in AHF patients (AUC = 0.691), which was superior to that of NT-proBNP (AUC = 0.545). The superior diagnostic performance of the combined panel (sST2 + AST + DBIL, AUC = 0.905) further suggests that sST2 adds unique information beyond conventional liver tests, possibly by capturing the inflammatory and fibrotic components of liver injury that are not fully reflected by transaminases alone. Nevertheless, our findings represent preliminary evidence of the association between sST2 and hepatic dysfunction, and the underlying mechanisms require further investigation in larger, mechanistic studies.

Despite these insights, our study has several limitations. First, this was a single-center study with a relatively small sample size, and the cross-sectional design precludes causal inference. In addition, the absence of follow-up observations—including dynamic changes in sST2 after discharge, recovery of liver function injury, and clinical outcomes such as mortality and readmission—precludes clarification of the prognostic value of sST2 in AHF patients with liver injury and verification of the causal relationship between sST2 and liver injury. Longitudinal and mechanistic studies are warranted to elucidate the temporal and causal relationship between sST2 elevation and the onset or progression of liver injury. Second, only 27 patients in our cohort met the diagnostic criteria for liver injury. This limited number may compromise the stability of the multivariate regression and ROC analyses and precluded meaningful subgroup stratification by liver injury severity (e.g., mild/moderate/severe). Additionally, within the liver injury group, the distribution of NYHA functional classes was highly unbalanced (22/27, 81.5% in class IV), which prevented reliable comparisons of sST2 levels across different NYHA classes (Figure 3C). Validation in larger, prospective multicenter cohorts is needed to confirm these associations, enable robust subgroup analyses, and strengthen generalizability. Third, the study did not include a control group of patients with isolated liver injury (without AHF). Due to ethical requirements and the need for additional protocol approval to enroll extra patients, we were unable to collect these data in the current study. Future studies should include such a control group to better differentiate the contributions of cardiac dysfunction and hepatic injury to sST2 elevation. Finally, tissue-level ST2 expression in the liver or myocardium was not directly evaluated in this study. Future research incorporating tissue or molecular analyses, as well as dynamic monitoring of sST2 responses to treatment, may help clarify the mechanistic pathways linking sST2 to cardiohepatic injury and its prognostic implications in HF.

## 5 Conclusion

In conclusion, our findings suggest that serum sST2 levels are significantly elevated in AHF patients with liver injury and are independently associated with liver function markers. These results suggest that sST2 may serve as a potential integrative biomarker reflecting both cardiac and hepatic dysfunction. Recognizing the broader systemic context of sST2 elevation in HF could help refine risk assessment and guide more comprehensive clinical management strategies. Although sST2 alone has limited sensitivity for liver injury, a combined panel of sST2, AST, and DBIL provides excellent diagnostic performance. This combined approach may serve as a useful tool for risk stratification and early identification of cardiogenic liver injury in AHF patients. Further research is warranted to elucidate the mechanistic underpinnings and prognostic implications of the cardiohepatic axis involving sST2.

## Data Availability

The datasets presented in this study can be found in online repositories. The names of the repository/repositories and accession number(s) can be found in the article/Supplementary Material.
