# Supplementary material for: Association of sST2 with liver function in patients with acute heart failure related hospitalization
Source: Front Cardiovasc Med. 2026 Sep 3;13:1781835. doi: 10.3389/fcvm.2026.1781835 (PMC13582450; doi:10.3389/fcvm.2026.1781835)
Supplement: Supplementary file 1 [file Datasheet1.docx]

Supplementary Material

## Supplementary Figures

**Supplementary Figure 1.** **Correlation analysis of NT-proBNP and liver function markers in HF patients.** (**A-F**) Scatter plots showing the relationships between serum NT-proBNP levels and liver injury parameters, including TBIL, DBIL, GGT, ALP, AST, and ALT, in 134 patients with heart failure. No significant correlations were observed between NT-proBNP and these liver function markers. The solid lines represent linear regression fits.

**Supplementary Figure 2. ROC analysis of sST2 between health control group and AHF patients.**

## Supplementary Tables

**Supplementary Table 1. Spearman's rank correlation coefficients between sST2 and various clinical and laboratory parameters.**

|  | **sST2** | | |
| --- | --- | --- | --- |
|  | Correlation coefficient | P value | n |
| Age | -0.092 | 0.290 | 134 |
| BMI | 0.101 | 0.346 | 90 |
| Hypertension | 0.001 | 0.992 | 134 |
| Diabetes mellitus | 0.038 | 0.662 | 134 |
| Valvular disease | -0.176 | 0.042 | 134 |
| Renal dysfunction | 0.083 | 0.340 | 134 |
| Arrhythmia | 0.087 | 0.320 | 134 |
| Venous thrombosis/intracardiac thrombosis | 0.161 | 0.063 | 134 |
| Acute coronary syndrome | 0.091 | 0.298 | 134 |
| Stroke | -0.049 | 0.574 | 134 |
| Liver injury | 0.265 | 0.002 | 134 |
| NYHA class | 0.131 | 0.131 | 134 |
| LVEF | 0.036 | 0.697 | 122 |
| NT-proBNP | 0.506 | <0.001 | 134 |
| ALP | 0.319 | <0.001 | 134 |
| ALT | 0.355 | <0.001 | 134 |
| AST | 0.396 | <0.001 | 134 |
| Cholesterol | -0.029 | 0.749 | 123 |
| Cr | 0.094 | 0.283 | 132 |
| Troponin I | 0.156 | 0.129 | 96 |
| DBIL | 0.314 | <0.001 | 134 |
| eGFR | -0.05 | 0.565 | 132 |
| GGT | 0.261 | 0.002 | 134 |
| GLU | -0.001 | 0.994 | 131 |
| HBA1C | 0.11 | 0.218 | 126 |
| HDL-C | -0.092 | 0.312 | 123 |
| LDL-C | -0.029 | 0.749 | 123 |
| NEU | 0.225 | 0.010 | 129 |
| PCT | 0.268 | 0.029 | 66 |
| TBIL | 0.243 | 0.005 | 134 |
| UA | 0.166 | 0.065 | 125 |
| WBC | 0.08 | 0.365 | 130 |

**Supplementary Table 2. Diagnostic performance of sST2, NT-proBNP, AST, DBIL, and sST2+AST+DBIL combination for predicting liver injury in patients with AHF.**

| **Variable** | **AUC** | **95% CI** | **Sensitivity** | **Specificity** |
| --- | --- | --- | --- | --- |
| sST2 | 0.691 | 0.605 - 0.768 | 37.00% | 99.10% |
| NT-proBNP | 0.545 | 0.457 - 0.631 | 77.80% | 37.40% |
| AST | 0.890 | 0.825 - 0.938 | 96.30% | 69.16% |
| DBIL | 0.858 | 0.787 - 0.912 | 81.50% | 81.30% |
| sST2+AST+DBIL | 0.905 | 0.842 - 0.949 | 85.20% | 85.00% |
